# Physiological Mechanism of Enhancing Salt Stress Tolerance of Perennial Ryegrass by 24-Epibrassinolide

**DOI:** 10.3389/fpls.2017.01017

**Published:** 2017-06-19

**Authors:** Wenli Wu, Qiang Zhang, Erik. H. Ervin, Zhiping Yang, Xunzhong Zhang

**Affiliations:** ^1^Institute of Agricultural Environment and Resources, Shanxi Academy of Agricultural SciencesTaiyuan, Shanxi, China; ^2^Department of Crop and Soil Environmental Sciences, Virginia Polytechnic Institute and State UniversityBlacksburg, VA, United States

**Keywords:** antioxidant, 24-epibrassinolide, hormones, salt stress, perennial ryegrass, ion

## Abstract

Brassinosteroids (BR) regulate plant tolerance to salt stress but the mechanisms underlying are not fully understood. This study was to investigate physiological mechanisms of 24-epibrassinolide (EBR)'s impact on salt stress tolerance in perennial ryegrass (*Lolium perenne* L.) The grass seedlings were treated with EBR at 0, 10, and 100 nM, and subjected to salt stress (250 mM NaCl). The grass irrigated with regular water without EBR served as the control. Salt stress increased leaf electrolyte leakage (EL), malondialdehyde (MDA), and reduced photosynthetic rate (Pn). Exogenous EBR reduced EL and MDA, increased Pn, chlorophyll content, and stomatal conductance (gs). The EBR applications also alleviated decline of superoxide dismutase (SOD) and catalase (CAT) and ascorbate peroxidase (APX) activity when compared to salt treatment alone. Salt stress increased leaf abscisic acid (ABA) and gibberellin A4 (GA4) content but reduced indole-3-acetic acid (IAA), zeatin riboside (ZR), isopentenyl adenosine (iPA), and salicylic acid (SA). Exogenous EBR at 10 nm and 100 nM increased ABA, and iPA content under salt stress. The EBR treatment at 100 nM also increased leaf IAA, ZR, JA, and SA. In addition, EBR treatments increased leaf proline and ions (K^+^, Mg^2+^, and Ca^2+^) content, and reduced Na^+^/K^+^ in leaf tissues. The results of this study suggest that EBR treatment may improve salt stress tolerance by increasing the level of selected hormones and antioxidant enzyme (SOD and CAT) activity, promoting accumulation of proline and ions (K^+^, Ca^2+^, and Mg^2+^) in perennial ryegrass.

## Introduction

Salinity is a major abiotic stress limiting growth and development of plants in many areas of the world due to increasing use of low quality of water for irrigation and soil salinization, and more than 20% of cultivated land worldwide (~45 hectares) is affected by salt stress and the amount is increasing (Gupta and Huang, 2014). Salinity stress may cause damage to plant physiological processes by over accumulation of reactive oxygen species (ROS), ion toxicity, impairment of antioxidant defense systems, photosynthetic function, and imbalance of hormones (Hu et al., 2012, 2015; Kim et al., 2016).

Previous studied have shown that salt stress may reduce stomatal conductance (gs) and inhibit gas exchange (Kim et al., 2016). The plants may suffer oxidative stress because of excess energy being directed to oxygen (O_2_), generating various ROS including superoxide (${\text{O}}_{2}^{-\cdot }$), hydrogen peroxide (H_2_O_2_), hydroxyl radicals (OH·), and singlet oxygen (^1^O_2_) (Hu et al., 2012). The ROS may damage cell membrane lipids, proteins, and nuclei acids, resulting in cell membrane electrolyte leakage (EL) and senescence (Huang et al., 2014). Plants have developed antioxidant defense mechanisms to eliminate ROS and prevent oxidative damage. Antioxidant enzymes, such as superoxide dismutase (SOD, EC 1.15.1.1), catalase (CAT, EC 1.11.1.6), and ascorbate peroxidase (APX, EC 1.11.1.11), protect plants against oxidative stress (Huang et al., 2014; Zhang et al., 2015). SOD constitutes the first line of defense against ROS by dismutating the superoxide anion to H_2_O_2_, which is finely regulated by CAT, POD, and APX (Gupta and Huang, 2014). Various antioxidant metabolites and enzymes may work coordinately in suppressing ROS toxicity under stressful environments.

Plants may accumulate various compatible solutes to cope with salt stress (Ghars et al., 2008; Khan et al., 2012; Kim et al., 2016). Proline severs as osmoprotentant and antioxidant to protect cells under salt stress. Salt stress may cause nutrient imbalance with increasing uptake of Na^+^ and decreasing K^+^, Mg^2+^, and Ca^2+^, resulting in Na^+^ toxicity (Hu et al., 2012; Gu et al., 2016).

Plant hormones may serve signals in regulating plant adaptation to salt stress environments (Strivastava, 2002; Ryu and Cho, 2015; Tang et al., 2015). The elevated ABA may help plants to acclimate under low water availability by closing stomata and accumulating compatible solutes for osmotic adjustment (Man et al., 2011; Zhang et al., 2015). Cytokinins (such as ZR and iPA) essentially regulate various plant developmental processes including cell division and enlargement, chloroplast biogenesis, nutrient mobilization, leaf senescence vascular differentiation and apical dominance (Ryu and Cho, 2015). Cytokinins facilitate the responses to delay both stomatal closure and leaf senescence under abiotic stresses (Ryu and Cho, 2015; Zhang et al., 2015). Auxin such as IAA can promote root initiation and also delay plant senescence (Zhang et al., 2009). Bioactive GAs such as GA4 are involve in plant growth and development such as leaf expansion stem elongation, and flowering (Ryu and Cho, 2015). There is cross-talking between GA action and other hormones signaling during abiotic stress to control plant growth and development (Ryu and Cho, 2015). JA is a critical signaling molecule for diverse developmental processes and defense responses in plants (Llanes et al., 2016). It was reported JA accumulated more in salt-tolerant cultivar than sensitive one in response to salt stress (Llanes et al., 2016). Recent studies have showed that SA is associated with plant tolerance salt stress and exogenous SA improved salt stress tolerance in various plant species (Khan et al., 2013; Song et al., 2014; Sun et al., 2015).

Brassinosteroids (BRs) are a new group of steroid hormones of plants, it is widely distributed in plant pollen, seeds, leaves, and other organs, and exhibit high physiological activity even at low concentrations (Bartwal et al., 2013; Sun et al., 2015). BRs are considered ubiquitous in plant kingdom as they found in almost all the phyla of the plant kingdom like alga, pteridophyte, gymnosperms, dicots, and monocots (Bajguz and Hayat, 2009). Previous studies have shown that exogenous application of BRs can affect a variety of physiological processes and improve plant tolerant to abiotic stresses, such as salt stress, drought stress, low and high temperature, and heavy metal stress (Shahbaz et al., 2008; Dalio et al., 2011; Vriet et al., 2012; Fariduddin et al., 2014; Sun et al., 2015; Vardhini and Anjum, 2015). 24-epibrassinolide (EBR), a highly active synthetic analog of BRs, is known to improve salt stress tolerance in perennial ryegrass (Sun et al., 2015), rice (Zdemir et al., 2004), and tomato (Ogweno et al., 2008). The physiological mechanisms of BRs' impact on plant tolerance to abiotic stress such as salt stress have not well documented. Vardhini and Anjum (2015) reviewed literatures showing that BRs may improve plant tolerance to abiotic stress mainly by enhancing antioxidant defense system. In salinity (120 mM) exposed IR-28 *Oryza sativa* seedlings, EBR considerably alleviated oxidative damage and improved seedling growth by increasing APX activity and reduced lipid peroxidation (Ozdemir et al., 2004). Sun et al. (2015) showed and application of EBR at 10 nM enhanced antioxidant enzyme activity in perennial ryegrass under salt stress. Shahid et al. (2011) reported that EBR treatment at 10 uM enhanced antioxidant activity, photosynthetic rate, stomatal conductance, and proline content in pea (*Pisum sativum* L.) under salt stress. Fariduddin et al. (2013) reported that EBR at 10 nM increased antioxidant and proline content in *Cucumis sativus* under salt stress. Plant hormones change in response to salt stress and EBR-induced increase in gs may be associated with alteration in hormonal balance. No research has been reported on EBR's effects on hormonal balance in turfgrass under salt stress.

As population and potable water demand increases, water shortage is a major problem in many parts of the world (Marcum and Pessarakli, 2010; Huang et al., 2014; Sun et al., 2015). Turfgrasses are increasingly experience salt stress due to the accelerated salinization of agricultural lands and increasing demand on use reclaimed or other secondary saline water for irrigation of turfgrass landscapes (Jiang et al., 2013; Huang et al., 2014; Sun et al., 2015). Perennial ryegrass (*Lolium perenne* L.) is one of the most important a cool-season grass species in temperate climate and widely used for home lawns, golf course, urban landscapes and other sports fields (Hu et al., 2012; Sun et al., 2015). It is also used for forage because of high yield and good quality (Hu et al., 2012). The objective of this study was to investigate physiological and metabolic responses of perennial ryegrass to EBR treatments under salt stress conditions and explore physiological mechanisms of EBR's impact on salt stress tolerance.

## Materials and methods

### Plant materials and culture

This study was conducted from April to June 2016 in the growth chamber facility at Virginia Tech, Blacksburg, VA, USA. Seeds of perennial ryegrass ‘Manhatan-5’ were obtained from Turf Merchants (Albany, OR, USA) were planted in plastic pot (16 cm diameter and 15 cm deep) filled with a fine sand containing 10% peat at a rate of 30 g m^−2^ pure live seeds on 27 Aril, 2016. A piece of plastic screen was placed in the bottom of the pot to prevent sand from leaching. The plants were grown in a growth chamber at temperatures (mean ± SD) at 22 ± 0.8/16 ± 0.6°C (day/night), 70 ± 8% relative humidity, 14-h photoperiod and photosynetically active radiation at 450 ± 11 umol m^−2^ s^−1^. The grass was fertilized at 1.5 g m^−2^ nitrogen from 28-8-18 complete fertilizer with micronutrients biweekly and trimmed to 6 cm weekly. The grass was irrigated by hand until water drained from bottom of the pots, three times per week.

### Treatments and sampling

Seventeen days after emergence, the grass was subjected to four treatments: (1) Control: normal water; (2) Salt Stress: 250 mM NaCl; (3) Salt stress (250 mM NaCl) plus 10 nM EBR (Sigma-Aldrich company, St. Louis, MO, USA); and (4) Salt stress (250 mM NaCl) plus 100 nM EBR. The EBR solution was sprayed to foliage using a hand-held sprayer at 10 mL per pot. After 12 h of EBR treatment, the salt solution was added in gradually increasing concentrations in aliquots of 50 mM every 12 h until the concentration of 250 mM was attained within 48 h after initiation and maintained concentrations by measuring the conductivity of the growth media (Shavrukov et al., 2012). The NaCl concentration (250 mM) selected based on our preliminary experiment and the previous studies (Hu et al., 2012; Sun et al., 2015) was suitable for perennial ryegrass. The EBR concentrations (10 and 100 nM) were used based on our preliminary screening study and previous studies (Shahid et al., 2011; Sun et al., 2015). Then each pot was placed in a plastic tray (20 cm diameter, 6.5 cm deep) filled with either salt treatment solution or distilled water only. The grass receiving the same volume of distilled water served as control. The lower one fifth portions of the pots were submerged in the solutions all the time. Stock solutions of EBR were prepared by dissolving the hormone in ethanol and stored at 4°C, the applied concentrations of EBR were prepared by diluting the stock solution with distilled water.

Leaf samples were collected at 0, 7, 14, 21, and 28 d after the initiation of salt treatment and a part of each sample was stored at −80^*o*^C for analysis of antioxidant enzyme, MDA, and various hormones. The rest of each sample was dried at 70°C for 3 days and used for analysis of ions. A small amount of fresh leaf tissues was collected for electrolyte leakage (EL) analysis.

### Measurements

#### Turfgrass quality

Turfgrass quality was visually rated based on a scale of 1–9 based on leaf color, uniformity, and density, with 1 indicating complete death or brown leaves, 6 representing minimum acceptable, and 9 indicating turgid and dark green leaves, with optimum canopy uniformity and density (Zhang et al., 2015).

#### Leaf electrolyte leakage (EL)

Fresh leaf (50 mg) were placed in closed test tube containing 10 mL deionized water and electrical conductance (EC1) was measured after shaken on a rotary shaker for 24 h at room temperature, then samples were subjected 120 °C for 30 in a autoclaved oven and electrical conductance (EC2) was measured again, the leaf electrolyte leakage was calculated as follows:

$$\begin{array}{l} \text{EL }\left(\text{\%}\right)\;=\;\left(\text{EC}1/\text{EC}2\right)\;\times \;100\text{\%} \end{array}$$

#### Leaf malondialdehyde (MDA)

Cell membrane lipid peroxidation was determined based on MDA content. The MDA was measured according to Hodges et al. (1999) with modifications. Leaf samples (50 mg) were homogenized in 1.8 ml 10% trichloroacetic acid (TCA) and centrifuged at 12,000 gn for 20 min. Then 1 ml 0.6% thiobarbituric acid (TBA) in 10% TCA was added to 1 ml supernatant. The mixture was heated in boiling water for 30 min then quickly cooled in an ice bath. After centrifugation at 1,600 gn for 10 min, the absorbance of the mixture was determined at 532 and 600 nm. Nonspecific absorbance at 600 nm was subtracted from that at 532 nm. The concentration of MDA was calculated using this adjusted absorbance and MDA's extinction coefficient of 155 mM^−1^cm^−1^.

#### Leaf chlorophyll (Chl) content

Leaf chl was extracted in acetone. Chlorophyll content was measured using a spectrophotometer according to Zhang et al. (2005).

#### Photosynthetic rate (Pn) and stomatal conductance (gs)

Leaf Pn and gs were measured using a portable photosynthetic system (LI-6400XT, Licor Corporation, Lincoln, Nebraska, USA). Four uniform leaf blades were sampled from each pot and placed in the gas chamber for measurement with settings of 22°C, CO_2_ flow rate at 400, CO_2_ concentration at 385 ppm, and PAR at 800 μmol m^−2^ s^−1^. Three readings from each sample were collected and the average was used for statistical analysis.

#### Leaf antioxidant enzyme activity

Frozen leaf samples (100 mg) were ground in liquid N2 and extracted in 1.8 ml of ice-cold 50 mmol sodium phosphate buffer (pH 7.0) containing 0.2 mM EDTA and 1% polyvinylpyrrolidone (PVP) in an ice-water bath. The homogenate was centrifuged at 12,000 gn for 20 min at 4°C. Supernatant was used to antioxidant enzyme activity.

Superoxide dismutase activity (SOD) was determined by measuring its ability to inhibit the photochemical reduction of nitro blue tetrazolium (NBT) according to the method of Giannopolitis and Ries (1977) with minor modifications. The reaction solution (1 ml) contained 50 mM phosphate buffer (pH 7.8), 0.1 mM EDTA, 13 mM methionine, 65 μM. NBT and 1.3 μM riboflavin, and 30 μL SOD extract. A solution containing no enzyme solution was used as the control. Test tubes were irradiated under fluorescent lights 60 μmol·m^−2^·s^−1^) at 25°C for 10 min. The absorbance of each solution was measured at 560 nm using a spectrophotometer, and one unit of enzyme activity was defined as the amount of enzyme that would inhibit 50% of NBT photoreduction.

Activities of catalase (CAT) was determined using the method of Chance and Maehly (1955) with modifications. For CAT, the reaction solution (1 mL) contained 50 mM phosphate buffer (pH 7.0), 15 mM H_2_O_2_, and 30 μL of extract. The reaction was initiated by adding the enzyme extract. Changes in absorbance at 240 nm were read in 1 min using a spectrophotometer (ε = 39.4 M^−1^cm^−1^).

The activity of ascorbate peroxidase (APX) was detected using the method of Zhang et al. (2015). The reaction solution (1 mL) contained 50 mM phosphate buffer (pH 7.0), 0.5 mM ascorbate, 0.1 mM EDTA and 100 μL enzyme extract. The reaction was started with addition of 10 μL 10 mM H_2_O_2_. The absorbance of the solution was determined at 290 nm after 1 min (ε = 2.8 mM^−1^cm^−1^).

#### Leaf hormone extraction and purification

Leaf hormones (IAA, ZR, iPA, ABA, SA, JA, and GA4) were extracted according to Edlund et al. (1995) and Zhang et al. (2009) with some modifications. Leaf tissues of each sample was ground with a mortar and pestle in liquid N_2_ to powder and the sample (50 mg) was extracted in 1.6 mL Na-phosphate buffer (0.05 M, pH 7.0) containing 0.02% sodium diethyldithiocarbamate as an antioxidant and the hormones were extracted for 1 h at 4°C with shaking. The C^13^-IAA (50 ng) was added into each sample as an internal standard. The pH of the samples was adjusted to 2.6 with 1.0 M HCl. The sample was slurried with 150 mg Amberlite XAD-7 (Sigma-Aldrich, St. Louis, MO, USA) for 30 min. After removal of the buffer, the XAD-7 was washed two times with 1 mL of 1% acetic acid before being slurried two times with 1.5 mL dichloromethane for 30 min each at 4°C (Edlund et al., 1995). The combined dichloromethane fractions were reduced to dryness with nitrogen gas. Then samples were dissolved in 210 μL methanol and diluted with 490 μL d.i. H_2_O containing 0.1% formic acid. The samples were filtered using an acrodisc 13-mm syringe filter with a 0.2-mm nylon membrane (Fisher Scientific Company, Pittsburgh, PA, USA.

#### Hormone analysis by LC-MS/MS

An Agilent tandem LC-MS/MS system with an ESI sample introduction interface (Agilent, Santa Clara, CA), consisting of 1,290 UPLC and 6490 QQQ, was used for analyzing IAA and ABA in extracts. The HPLC separation was performed on an Agilent Zorbax Extend-C18 analytical (4.6 × 50 mm, 5 μm) and guard (4.6 × 12 mm, 5 μm) columns. The analytes were eluted with water (mobile phase A) and methanol (B) in 0.1% formic acid in a gradient: 0–4.5 min B increasing from 30 to 80%, 4.5–5 min B increasing to 100%, 5–7 min B at 100%, and B decreasing to 30% at 7.5 min. The injecting volume was 10 μL and flow rate was 0.5 mL·min^−1^. The column temperature was 40°C. The chromatography retention time (RT), precursor ion, fragmental reactions monitored, ionization mode, and collision energies (CE) used for each compound are given in Table 1. The C^13^-labeled IAA (IAA_d5_) was used as an internal standard. The source parameters were: nebulizer pressure 310 kPa, dry gas temperature 250°C, sheath gas temperature 200°C, and gas flow 8 mL min^−1^. The selected hormones were determined based on retention times and ion products and standards of each compound.

**Table 1:** The parameters for analysis of leaf indole-3-acetic acid (IAA), C13-labeled IAA, abscisic acid (ABA), *trans*-zeatin riboside (t-ZR), isopentenyl adenosine (iPA), gibberellin A4 (GA4), salicylic acid (SA), and jamonic acid (JA) of perennial ryegrass using liquid chromatography-mass spectrometry (LC-MS/MS).

**Analyte**	**Retention Time (min)**	**Precursor Ion**	**Production**	**Application**	**Collision Energy (V)**	**Mode**
IAA	3.9	176.1	130.2	Quantitative	12	+
			77.2	Qualitative	52	+
C^13^-IAA	3.9	182.1	136.2	Quantitative	12	+
			83.2	Qualitative	52	+
ABA	4.6	265.2	229.2	Quantitative	0	+
			91.2	Qualitative	4	+
t-ZR	2.3	352.2	220	Quantitative	16	+
			136.2	Qualitative	32	+
Ipa	4.3	336	204	Quantitative	20	+
			136	Qualitative	30	+
GA4	6	331.3	213.2	Quantitative	35	−
			269.1	Qualitative	20	−
SA	4.7	137	93.1	Quantitative	13	−
			64.9	Qualitative	33	−
JA	5.3	209.1	59.1	Quantitative	9	−
			165.1	Qualitative	5	−

#### Leaf proline content

Proline content was determined with the method of Bates et al. (1973) with some modifications. Briefly, leaf (50) were homogenized with 1.8 mL 3% sulfosalicylic acid and boiling at 100°C for 10 min, 1 mL of the supernatant was mixed with 1 mL acetic acid and 1 mL acidic ninhydrin and heated at 100°C for 40 min, the reaction mixture was extracted with 2 mL toluene after cooling and read the absorbance at 520 nm.

#### Leaf ion content

The contents of Na^+^, K^+^, Ca^2+^, and Mg^2+^ in the leaf samples were measured by the methods of Jones et al. (1991) with some modifications. The dried leaves (100 mg) were weighed and ash in the muffle furnace at 490°C for 8 h, the ashes were dissolved with 2 mL 1 M HCl and then diluted to 10 mL with d.i. H_2_O. The concentrations of ion elements were determined by using an inductively coupled plasma mass spectrometer (Thermo Scientific, Waltham, MA).

#### Experimental design and statistical analysis

The four treatments were arranged in a completely randomized block design with four replications. The data were subjected to one-way analysis of variance using SAS software (v. 9.3 for Window; SAS Institute, Cary, NC, USA, 2010). The mean separations were performed using the Fisher's protected least significant difference test at the *P* = 0.05 probability level.

## Results

### Turf quality

**Figure 1:**
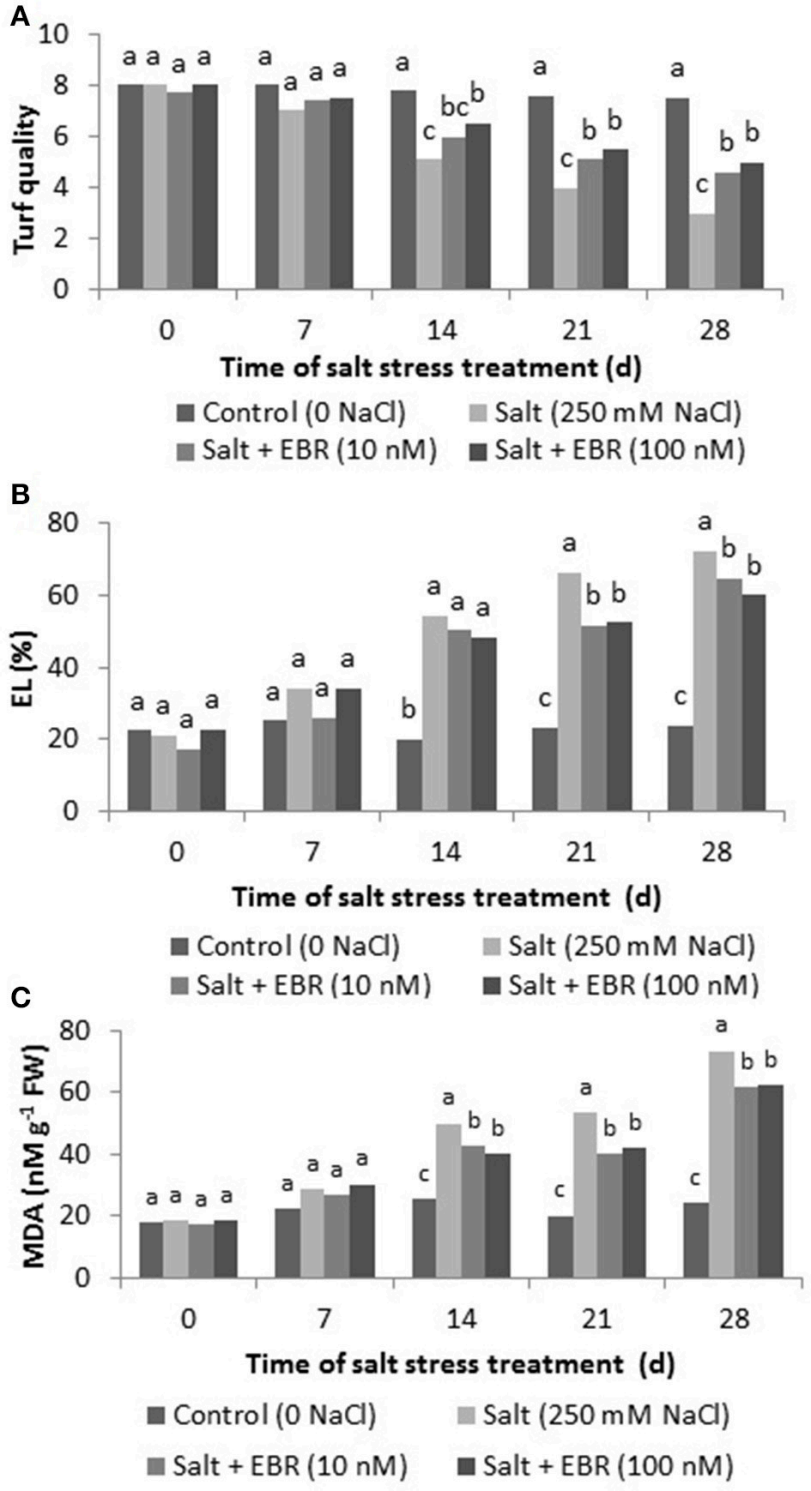
Effects of 24-epibrassinolide on turf quality **(A)**, leaf electrolyte leakage (EL, **B**), malondialdehyde (MDA, **C**) in perennial ryegrass under salt stress. Treatments with same letters for each sampling date are not significantly different at *P* = 0.05.

Salt stress reduced turf quality at days 14, 21, and 28. The EBR treatments alleviated turf quality decline as measured at days 14, 21, and 28 (Figure 1A). At day 28, EBR treatments at 10 nM and 100 nM increased turf quality rating by 53.3 and 66.7%, respectively, when compared to salt stress alone.

### Leaf electrolyte leakage (EL)

Salt stress increased EL as measured on days 14, 21, and 28 (Figure 1B). The EBR application at 10 and 100 nM reduced EL as measured at days 21 and 28. At day 28 of salt stress, EBR at 10 and 100 nM reduced EL by 10.5 and 17.0%, respectively, when compared to the salt treatment alone.

### Leaf malondialdehyde (MDA) content

Salt stress increased MDA content relative to the control (no salt stress) as measured on days 14, 21, and 28 (Figure 1C). The EBR application at 10 and 100 nM reduced MDA relative to salt stress treatment as measured at days 21 and 28. On day 28 of salt stress, EBR at 10 and 100 nM reduced EL by 10.5 and 17.0%, respectively, when compared to the salt treatment alone.

### Leaf chl content

**Figure 2:**
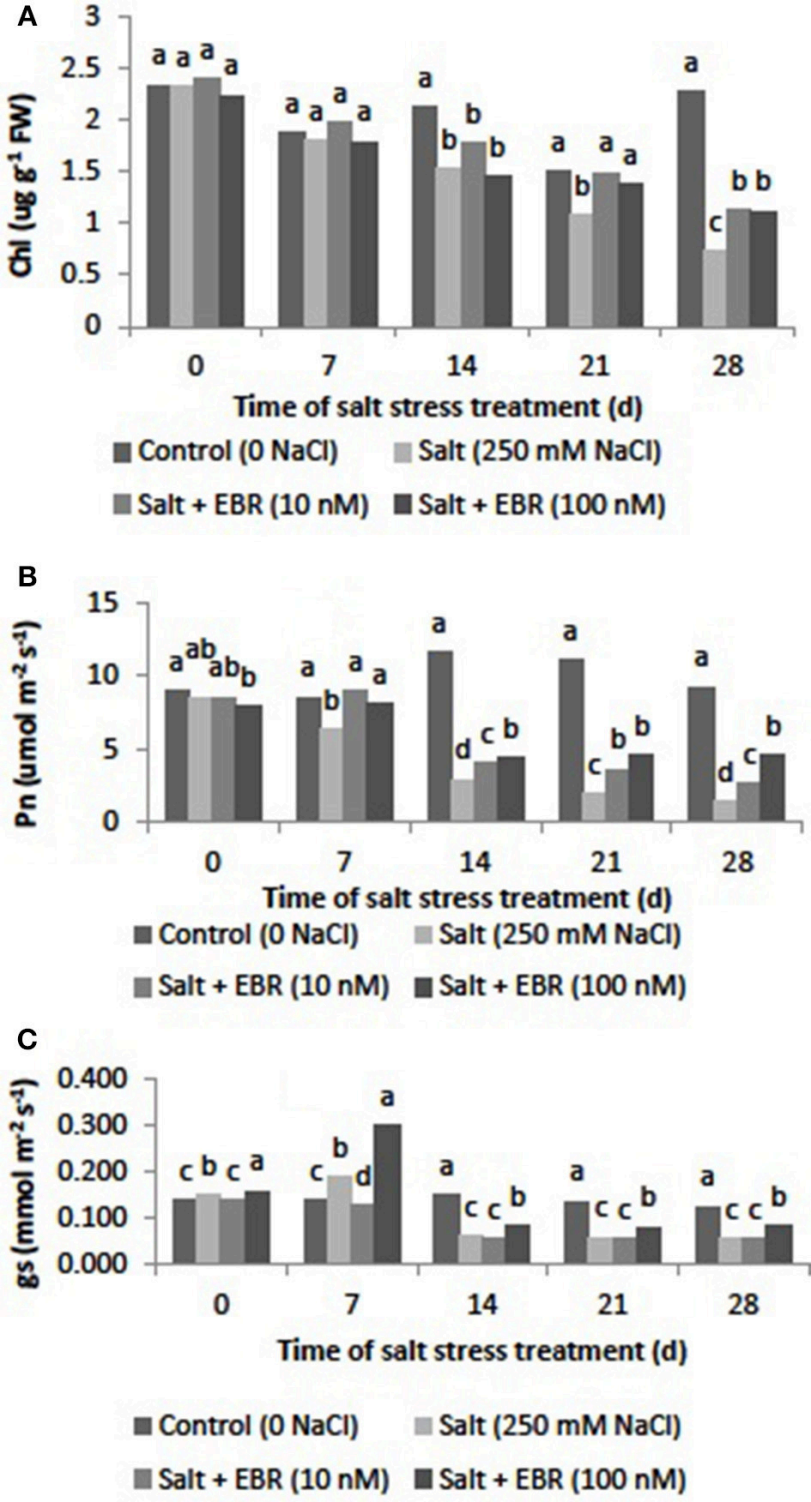
Effects of 24-epibrassinolide on leaf chlorophyll content (**A**, photosynthetic rate (Pn, **B**) and stomatal conductance (gs, **C**) of perennial ryegrass under salt stress. Treatments with same letters for each sampling date are not significantly different at *P* = 0.05.

Salt stress reduced chl content at days 14, 21, and 28. The EBR treatments alleviated chl decline at days 21 and 28 (Figure 2A). At day 28, EBR treatments at 10 and 100 nM increased chl content by 52.7 and 51.4%, respectively, when compared to salt stress alone.

### Leaf photosynthetic rate (Pn) and stomatal conductance (gs)

Salt stress reduced leaf Pn relative to the control beginning 7 d of salt stress (Figure 2B). At day 28, salt stress reduced Pn by 70.7%. Application of EBT at 10 and nM alleviated Pn decline as measured at days 14, 21, and 28. The EBR at 10 and 100 nM increased Pn by 0.8 and 2.1-fold relative to the salt treatment.

Salt stress reduced leaf gs relative to the control beginning at 7 d of salt stress (Figure 2C). Application of EBR at 10 and 100 nM alleviated gs decline as measured at days 7, 14, 21, and 28.

### Leaf superoxide dismutase (SOD) catalase (CAT), and ascorbate peroxide (APX) activity

**Figure 3:**
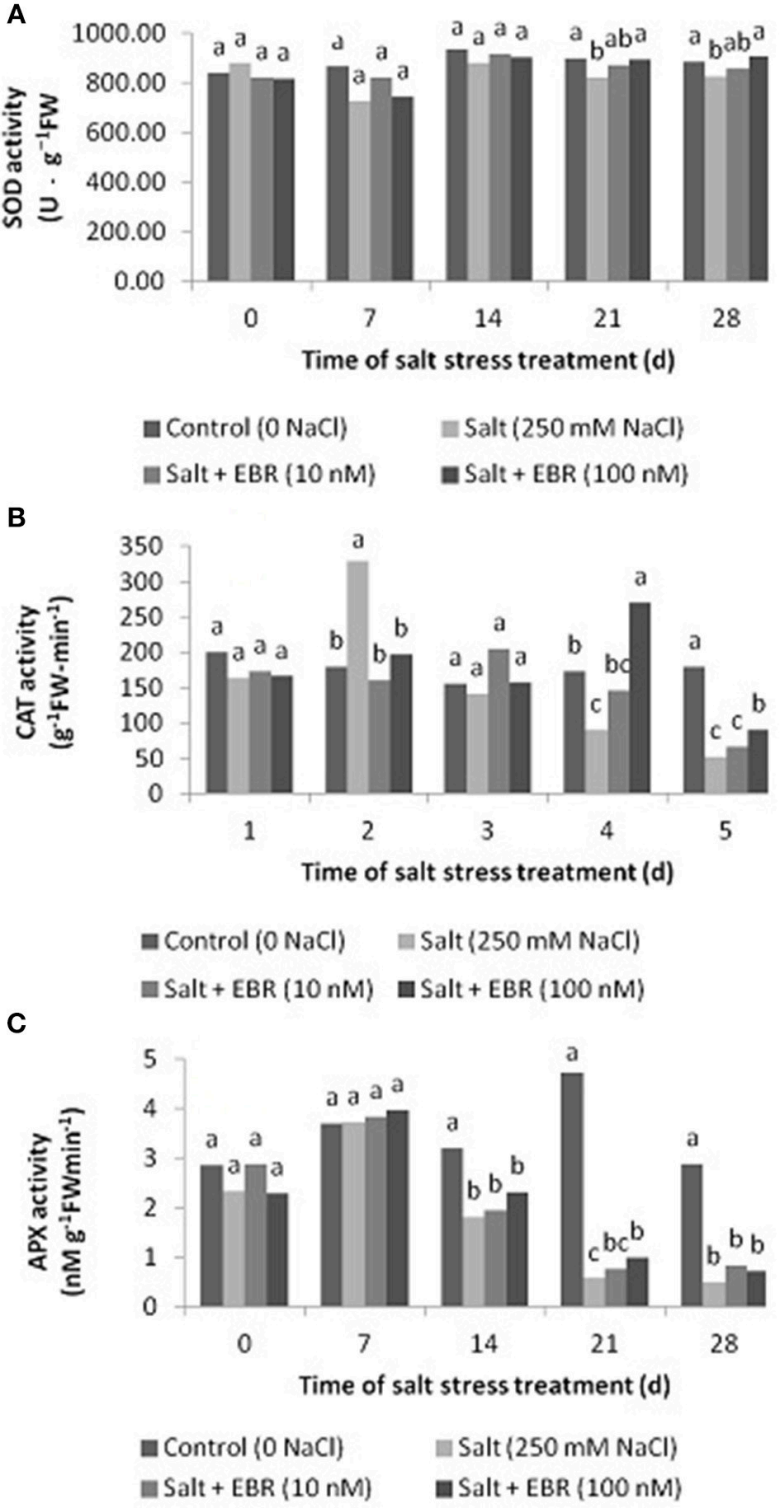
Effects of 24-epibrassinolide on leaf superoxide dismutase (SOD, **A**), catalase (CAT, **B**) and ascorbate peroxidase (APX, **C**) activity of perennial ryegrass under salt stress. Treatments with same letters for each sampling date are not significantly different at *P* = 0.05.

Salt stress reduced SOD activity as measured at days 21 and 28 (Figure 3A). Application of EBR at 100 nM alleviated SOD decline as measured at days 21 and 28. No SOD difference between treatments were found at days 0, 7, and 14. Similar to SOD, salt stress reduced CAT activity as measured at days 21 and 28 (Figure 3B). EBR treatment at 100 nM increased CAT activity relative to the salt stress alone at days 21 and 28.

Salt stress reduced APX activity as measured at days 14, 21, and 28 (Figure 3C). The EBR treatment at 100 nM alleviated APX decline as measured at day 21 only.

### Leaf indole-3-acetic acid (IAA) and abscisic acid (ABA) content

**Figure 4:**
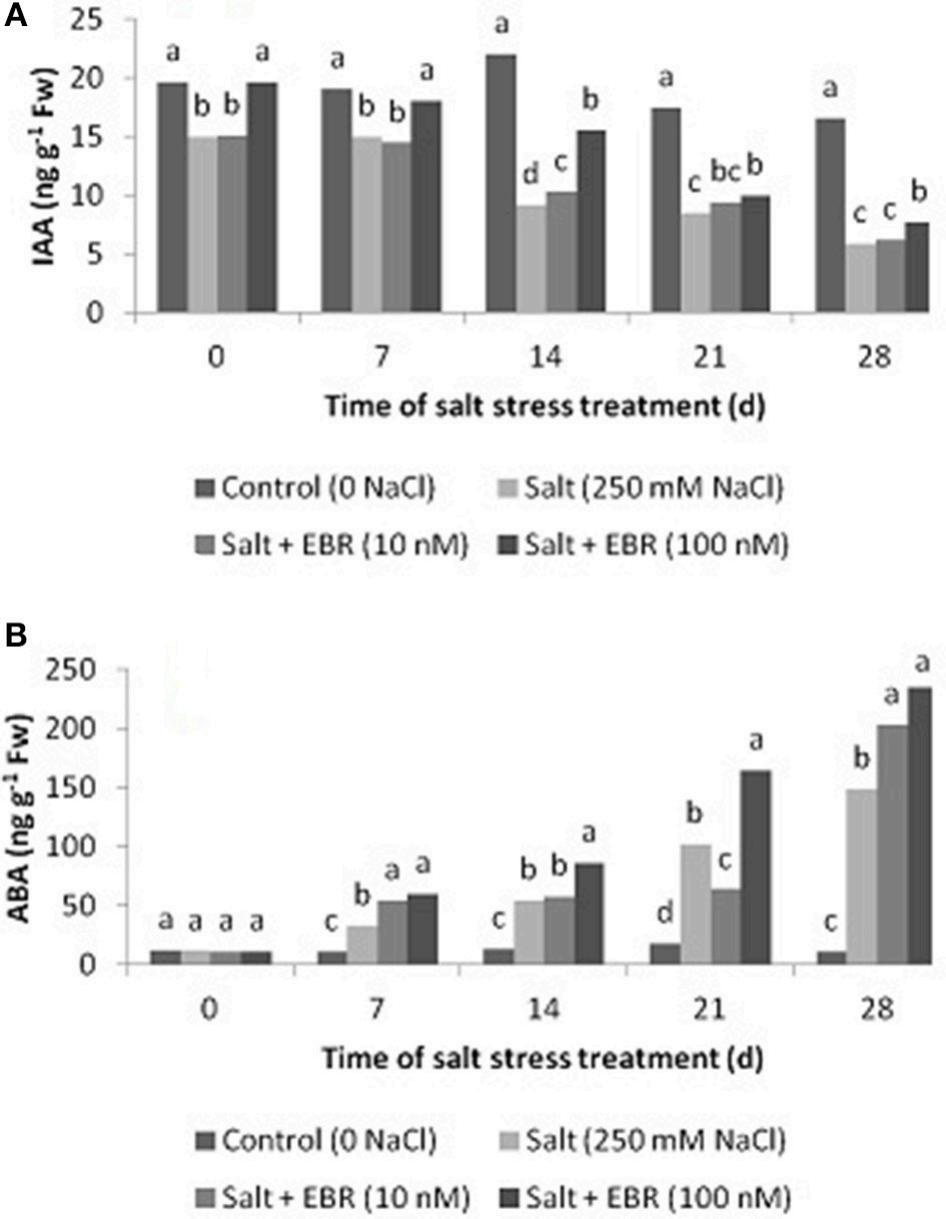
Effects of 24-epibrassinolide on leaf indol-3-acetic acid (IAA, **A**), and abscisic acid (ABA, **B**) of perennial ryegrass under salt stress. Treatments with same letters for each sampling date are not significantly different at *P* = 0.05.

Salt stress reduced leaf IAA as measured at days 14, 21, and 28 (Figure 4A). The EBR at treatment at 100 nM increased leaf IAA relative to the salt stress alone at days 7, 14, 21, and 28. The EBR treatment at lower rate (10 nM) also increased IAA content at day 14.

Salt stress induced accumulation of ABA in leaf tissues as measured at days 7 through 28 (Figure 4B). The EBR treatment at 100 nM increased ABA content from days 7 through 28. The EBR at 10 nM also increased ABA content at days 7 and 28 when compared to the salt stress treatment alone. At day 28, the EBR treatments at 10 and 100 nM increased ABA content by 37.2 and 58.6%, respectively, when compared to salt stress alone.

### Leaf zeatin riboside (ZR) and isopentenyl adenosine (iPA)

**Figure 5:**
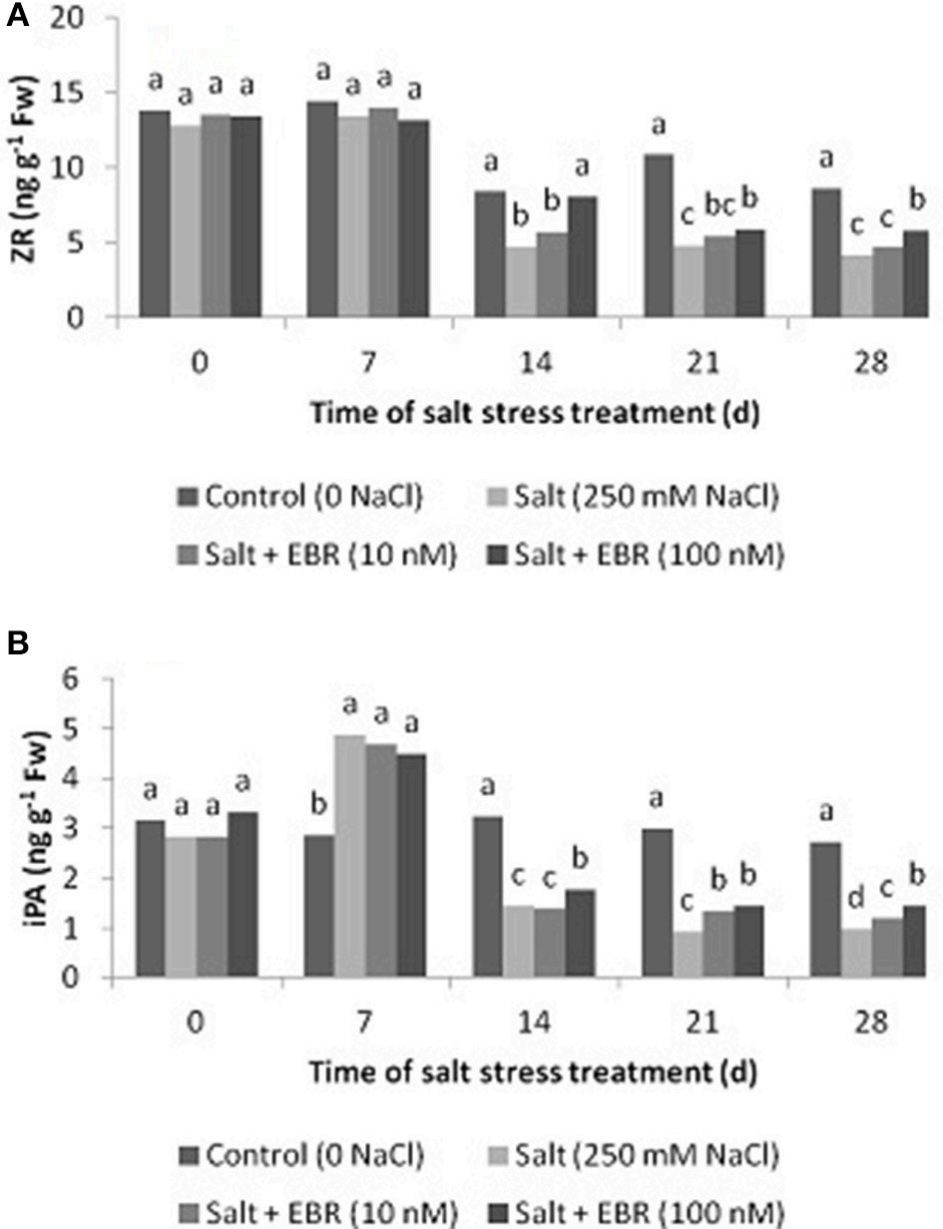
Effects of 24-epibrassinolide on leaf zeatin riboside (ZR, **A**), isopentenyl adenosine (iPA, **B**) of perennial ryegrass under salt stress. Treatments with same letters for each sampling date are not significantly different at *P* = 0.05.

Salt stress reduced leaf ZR content at days 14, 21, and 28 (Figure 5A), and also reduced iPA content at day 28 (Figure 5B). Application of EBR at 100 nM increased ZR relative to salt stress treatment alone as measured at days 14, 21, and 28. At day 28, EBR treatment at 100 nM increased ZR by 39.8%. Similarly, EBR treatment at 100 nM increased iPA at days 14, 21, and 28. In addition, EBR at 10 nM also increased iPA content at days 21 and 28. At day 28, EBR treatments at 10 and 100 nM increased iPA content by 23.5 and 48.0%, respectively, when compared to the salt treatment alone.

### Leaf salicylic acid (SA), jasmonic acid (JA), and gibberellin A4 (GA4)

**Figure 6:**
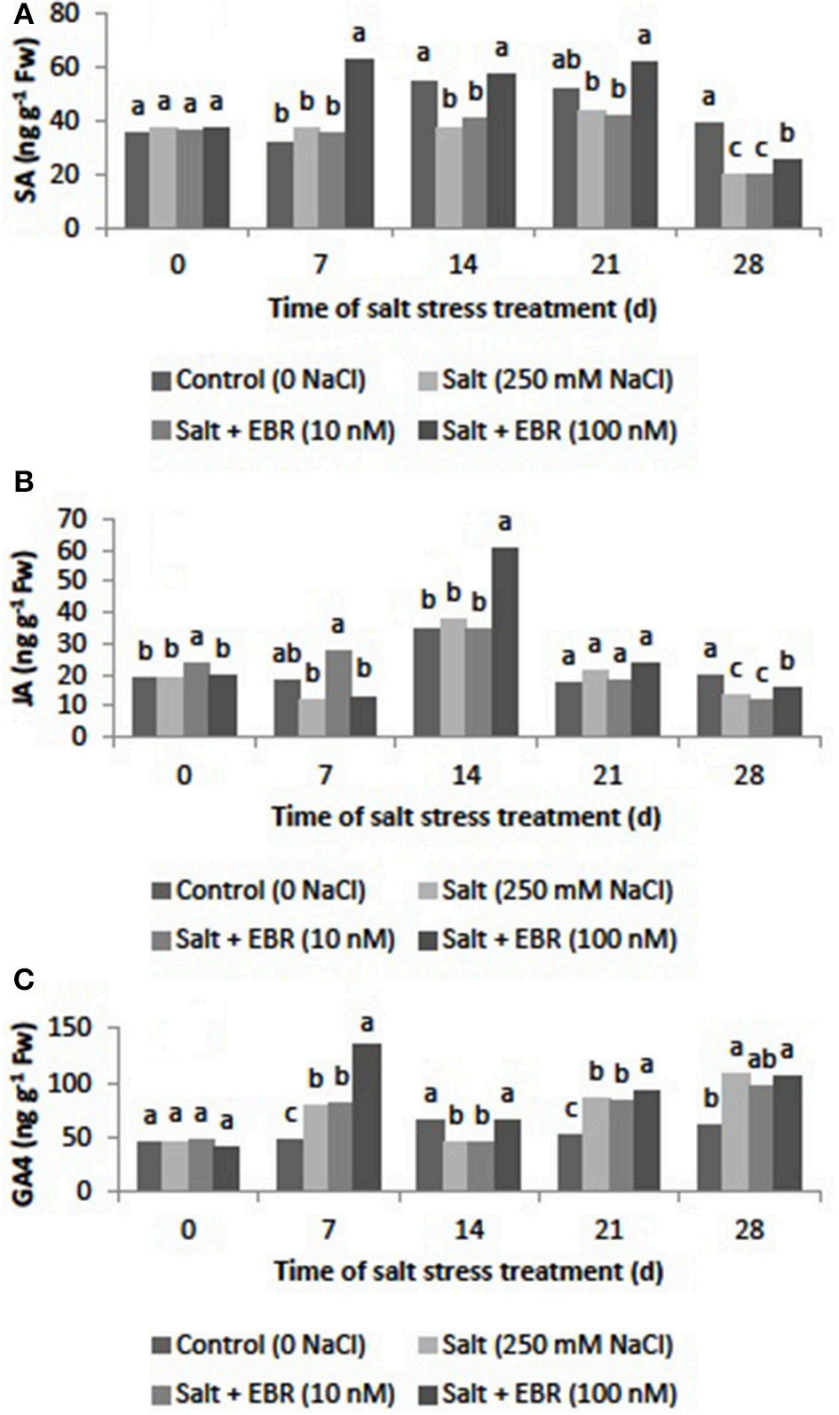
Effects of 24-epibrassinolide on leaf salicylic acid (SA, **A**), jasmonic acid (JA, **B**), and gibberellin A4 (GA4, **C**) of perennial ryegrass under salt stress. Treatments with same letters for each sampling date are not significantly different at *P* = 0.05.

Salt stress reduced SA content as measured at days 14 and 28 (Figure 6A). The EBR treatments at 100 nM increased SA content relative to the salt stress alone at days 7, 14, 21, and 28. At day 28, the EBR treatment at 100 nM increased SA by 24.8% when compared to the salt stress treatment alone.

Salt stress reduced leaf JA content as measured at day 28 (Figure 6B). Application of EBR at 100 nM increased JA content relative to the salt stress treatment alone at day 28, when the EBR at 100 nM increased JA content by 16.8%.

GA4 increased in responses to salt stress as measured at days 7, 21, and 28 (Figure 6C). The EBR treatment at 100 nM increased GA4 when compared to the sat stress treatment alone at days 7, 14, and 21.

### Leaf proline content

As measured at day 28, salt stressed grass contained higher levels of proline in leaf tissues than no-salt control (Table 2). The EBR treatment at 10 and 100 nM increased proline content by 31.0- and 29.5-fold, respectively, relative to the salt stress treatment only.

**Table 2:** Leaf proline content and ion content responses to 24-epibrassinolide (EBR) in perennial ryegrass under salt stress.

**Treatment**	**Proline**	**Ion content (mg g^−1^ DW)**				
	**(μg g^−1^ FW)**	**Na^+^**	**K^+^**	**Ca^2+^**	**Mg^2+^**	**Na^+^/K^+^**
Control (0 NaCl)	121.0c	5.6b	21.63a	11.58a	2.73a	0.26d
Salt (250 mM NaCl)	2,373.4b	89.05a	10.85d	5.55c	1.52c	8.223a
Salt + EBR (10 nM)	3,748.5a	84.95a	13.75c	6.73b	2.15b	6.181b
Salt + EBR (100 nM)	3,564.4a	82.50a	15.55b	6.43b	2.10b	5.309c

*Means followed by same letters within same column are not significantly different at P = 0.05*.

### Leaf ion content

At day 28, salt stress treatment increased Na^+^ content but reduced K^+^, Mg^2+^, and Ca^2+^ content in leaf tissues when compared to no-salt control (Table 2). The EBR treatments at 10 nM increased K^+^, Ca^2+^, and Mg^2+^ content, did not impact on Na^+^, and reduced Na^+^/K^+^ ratio. The EBR treatments at 10 and 100 nM reduced Na^+^/K^+^ by 24.8 and 35.4%, respectively, when compared to the salt stress treatment alone.

## Discussion

The results of this study showed that salt stress at 250 mM NaCl caused significant damage to perennial ryegrass. The salt stress caused lipid peroxidation (higher MDA), damaged cell membrane (higher EL), and photosynthetic function (lower chl, Pn, and gs). Application of EBR at 10 and 100 nM alleviated salt-induced injury of cell membrane and photosynthetic function. This is in general agreement with previous studies by Ding et al. (2012) with eggplants (*Disambigutation* L.) and Ozdemir et al. (2004) with rice (*Oryza sativa* L.). Hu et al. (2013) reported that stress may cause damage to perennial ryegrass through stomatal limitation. Salt-induced osmotic stress causes stomatal closure and inhibits gas exchange, resulting in lipid peroxidation and cell membrane damage due to ROS accumulation. Our results showed that EBR treatments increased gs and Pn, suggesting that EBR may improve gas exchange and cell membrane integrity.

Plants possess antioxidant defense systems to cope with ROS induced by salt stress (Gupta and Huang, 2014). The results of this study indicated that salt stress reduced antioxidant enzyme activity, especially CAT and APX (Figure 3). Application of EBR at 100 nM improved SOD and CAT activity under salt stress. The EBR at 100 nM also increased APX activity at day 21. This is consistent with previous studies (Shang et al., 2006; Shahbaz and Ashraf, 2007; Sharma et al., 2013). Several studies have showed that application of BR had a positive effect on antioxidant enzymes to protect plant under stress condition (Alscher et al., 2002; Ogweno et al., 2008). Bajguz and Hayat (2009) found that treatment with EBR increased CAT and APX activities in green algae (*Chlorella vulgaris* L.). Shang et al. (2006) reported that exogenous BR protected Cucumber (*Cucumis sativus* L.) seedlings against salt stress by elevating the activity of SOD, CAT, and POD. Sun et al. (2015) noted that EBR at 10 nM reduced ROS and lipid peroxidation, increased SOD, CAT, POD, and APX activity and improved salt stress tolerance in perennial ryegrass. These results suggest that EBR-induced salt stress tolerance improvement may be closely related to its enhancement of antioxidant defense system.

Plant hormones play an important in regulating plant tolerance to salt stress (Ryu and Cho, 2015). The results of this study indicated that salt stress increased ABA and GA4 content but reduced levels of IAA, ZR, iPA, SA, and JA. Application of EBR alleviated decline of these hormones and promoted ABA content (Figures 4–6). It has been documented that elevated ABA may induce stomatal closure (Zhang et al., 2015). Cytokinins and IAA may delay leaf senescence and improve photosynthetic function. Recent studies have showed that SA is closely associated with salt stress tolerance in plants (Davies, 2010; Khan et al., 2013; Song et al., 2014). The JA could be act as an effective protector against salt-mediated adverse effects (Ryu and Cho, 2015). Maggio et al. (2010) found that exogenous GA3 enhanced water availability at low salt stress conditions. Our results suggest that EBR application may improve photosynthetic function and salt stress tolerance by increasing the levels of the selected hormones, especially ZR, iPA, IAA, and SA, increasing gas exchange (higher gs), and delaying plant senescence.

The results of this study indicated that EBR treatment increased leaf proline content. This is consistent with previous studies by Sun et al. (2015), Shahid et al. (2011), and Shang et al. (2006). Proline functions as an osmoprotectant and ROS scavenger (Ghars et al., 2008; Kim et al., 2016). The elevated proline due to EBR treatment will facilitate osmotic adjustment and improve plant tolerance to salt stress.

Maintaining ion homoeostasis by ion uptake and compartmentalization is not only crucial for normal plant growth but also is essential process for growth during salt stress (Shahbaz and Ashraf, 2007; Ghars et al., 2008; Gupta and Huang, 2014). Our results showed that EBR treatment increased K^+^, Ca^2+^, and Mg^2+^ content and reduced Na^+^/K^+^ ratio in perennial ryegrass under salt stress. The EBR treatment at 10 and 100 nM reduced Na^+^/K^+^ by 24.8 and 35.4%, respectively, when compared to the salt stress treatment alone. This is consistent with previous study by the Sun et al. (2015) who reported that EBR treatment at 10 nM reduced leaf Na^+^/K^+^ and increased K^+^, Mg^2+^, and Ca^2+^ content in perennial ryegrass under salt stress. Excess Na^+^ in leaf tissues may perturb metabolic processes. The high Na^+^ concentration, resulting in the decreased K^+^, Ca^2+^, Mg^2+^ influx and increased Na^+^ influx, may cause ion toxicity and osmotic stress that seriously affect the normal growth of plants (Fariduddin et al., 2013; Sun et al., 2015). Exogenous EBR could ameliorated ion toxicity and nutritional imbalance under salt stress by regulating ion balance and osmotic adjustment.

**Figure 7:**
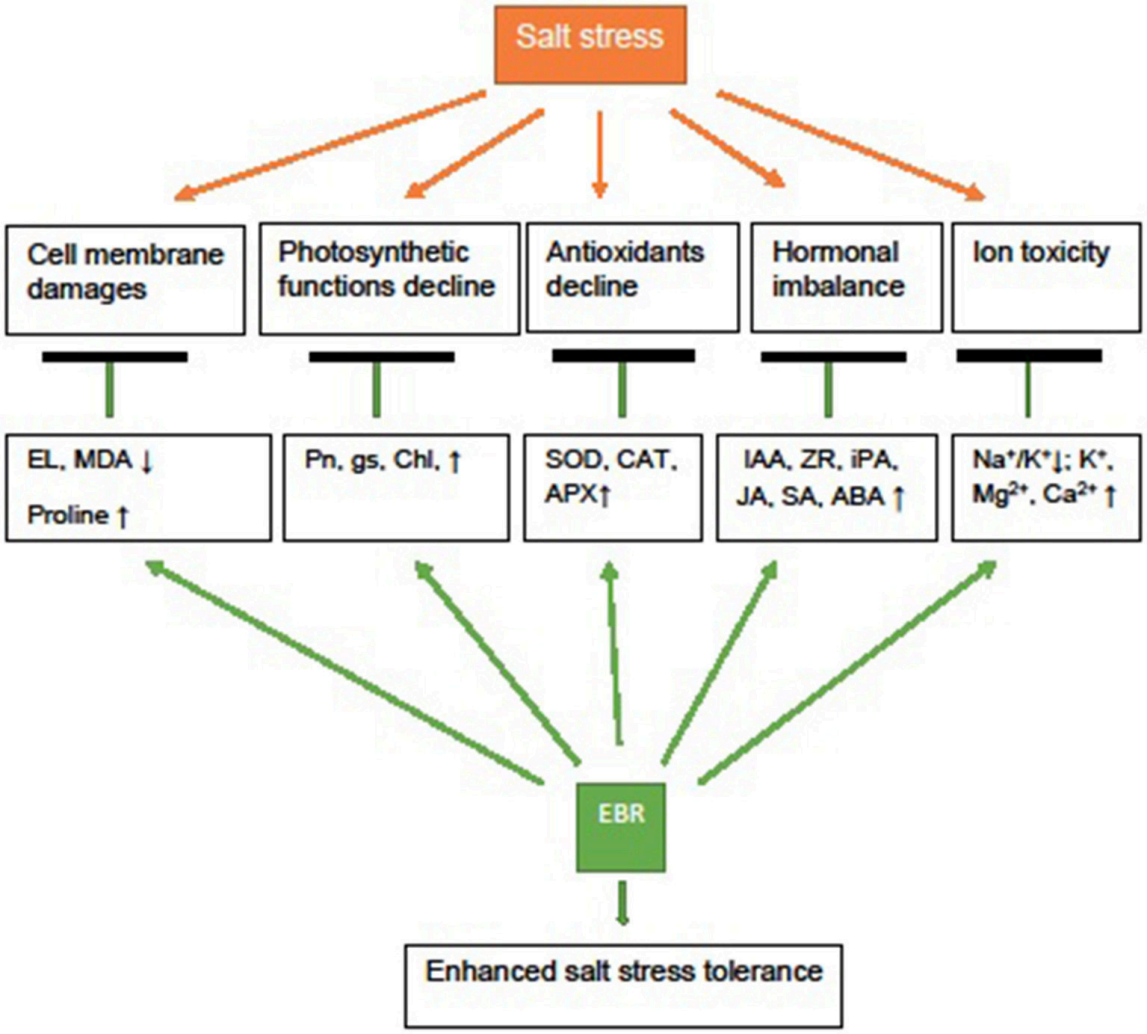
Possible mechanism depicting 24-epibrassinolide (EBR) application involved in salt stress tolerance of perennial ryegrass. Exogenous EBR could improve cell membrane integrity and stabilization (lower MDA, EL, higher proline), improve photosynthetic function (higher Pn, gs, and chl), antioxidant defense (higher SOD, CAT, and APX activity), hormonal metabolism (higher IAA, ZR, iPA, JA, SA, and ABA) and reduced ion toxicity (lower Na+ and Na^+^/K^+^, higher K+, Mg^2+^, and Ca^2+^), resulting in enhanced salt stress tolerance and visual quality.

In summary, salt stress (250 mM NaCl) caused lipid peroxidation, and damaged cell membrane, photosynthetic function, increased Na^+^ accumulation, and suppressed anti-senescence hormones, especially ZR, iPA, and IAA, and reduced visual quality in perennial ryegrass. Application of EBR, especially at 100 nM, improved cell membrane integrity, Pn, and gas exchange (higher gs) and protected chl. The EBR application also increased antioxidant enzyme (SOD, CAT, and APX) activity, alleviated decline of plant hormones (IAA, ZR, iPA, JA, SA, ABA), increased proline and ions (K^+^, Ca^2+^, and Mg^2+^) (Figure 7). The shift of hormonal balance by EBR may signal antioxidant defense and osmotic adjustment responses, delaying leaf senescence and protecting photosynthetic function under salt stress. The results of this study suggest that EBR treatment may improve salt stress tolerance of perennial ryegrass by increasing the level of elected hormones, antioxidant enzyme (SOD, CAT, and APX) activity, proline, and promoting uptake of ions (K^+^, Ca^2+^, and Mg^2+^) to reduce Na^+^/K^+^, resulting in improved cell membrane integrity, photosynthetic function and visual quality in perennial ryegrass.

## Author contributions

Conceived and designed the experiments: XZ, EE, and WW. Performed the experiments: WW and XZ. Analyzed the data: XZ, WW, and QZ. Wrote the paper: WW, XZ, QZ, EE, and ZY.

### Conflict of interest statement

The authors declare that the research was conducted in the absence of any commercial or financial relationships that could be construed as a potential conflict of interest.

## References

[B1] Alscher R. G. Erturk N. Heath L. S. (2002). Role of superoxide dismutases (SODs) in controlling oxidative stress in plants. J. Exp. Bot. 53, 1331–1341. 10.1093/jxb/53.372.1331 11997379

[B2] Bajguz A. Hayat S. (2009). Effects of brassinosteroids on the plant responses to environmental stresses. Plant Physiol. Biochem. 47, 1–8. 10.1016/j.plaphy.2008.10.002 19010688

[B3] Bartwal A. Mall R. Lohani P. Guru S. K. Arora S. (2013). Role of secondary metabolites and brassinosteroids in plant defense against environmental stresses. J. Plant Growth Regul. 32, 216–232. 10.1007/s00344-012-9272-x

[B4] Bates L. S. Waldren R. P. Tear I. D. (1973). Rapid determination of free proline for water-stressed studies. Plant Soil 39, 205–207. 10.1007/BF00018060

[B5] Chance B. Maehly A. C. (1955). Assay of catalases and peroxidases. Methods Enzymol. 2, 764–775. 10.1016/S0076-6879(55)02300-8 13193536

[B6] Dalio R. J. D. Pinheiro H. P. Sodek L. Haddad C. R. B. (2011). The effect of 24-epibrassinolide and clotrimazole on the adaptation of *Cajanus cajan* (L.) Millsp. to salinity. Acta Physiol. Plant. 33, 1887–1896. 10.1007/s11738-011-0732-x

[B7] Davies P. J. (2010). Plant Hormones: Biosynthesis, Signal Transduction, Action, 3rd Edn. New York, NY: Springer.

[B8] Ding H. D. Zhu X. H. Zhu Z. W. Yang S. J. Zha D. S. Wu X. X. (2012). Amelioration of salt-induced oxidative stress in eggplant by application of 24-epibrassinolide. Biol. Plant. 56, 767–770. 10.1007/s10535-012-0108-0

[B9] Edlund A. Eklof S. Sundberg B. Moritz T. Sandberg G. (1995). A microscale technique for gas chromatography-mass spectrometry measurements of pictogram amounts of indole-3-aceticv acid in plant tissues. Plant Physiol. 108, 1043–1047. 10.1104/pp.108.3.1043 12228526 PMC157455

[B10] Fariduddin Q. Khalil R. R. A. E. Mir B. A. Yusuf M. Ahmad A. (2013). 24-Epibrassinolide regulates photosynthesis, antioxidant enzyme activities and proline content of Cucumis sativus under salt and/or copper stress. Environ. Monit. Assess. 185, 7845–7856. 10.1007/s10661-013-3139-x 23443638

[B11] Fariduddin Q. Yusuf M. Ahmad I. Ahmad A. (2014). Brassinosteroids and their role in response of plants to abiotic stresses. Biol. Plant. 58, 9–17. 10.1007/s10535-013-0374-5

[B12] Ghars M. A. Parre E. Debez A. Abdelly C. (2008). Comparative salt tolerance analysis between *Arabidopsis thaliana* and *Thellungiella halophila*, with special emphasis on K^+^/Na^+^ selectivity and proline accumulation. J. Plant Physiol. 165, 588–599. 10.1016/j.jplph.2007.05.014 17723252

[B13] Giannopolitis C. N. Ries S. K. (1977). Superoxide dismutase occurrence in higher plants. Plant Physiol. 59, 309–314. 10.1104/pp.59.2.309 16659839 PMC542387

[B14] Gu M. F. Li N. Shao T. Y. Long X. H. Brestic M. Shao H. B. (2016). Accumulation capacity of ions in cabbage (*Brassica oleracea* L.) supplied with sea water. Plant Soil Environ. 62, 314–320. 10.17221/771/2015-PSE

[B15] Gupta B. Huang B. (2014). Mechanism of salinity tolerance in plants: physiological, biochemical, and molecular characterization. Intl. J. Genomics. 2014:701596. 10.1155/2014/701596 24804192 PMC3996477

[B16] Hodges D. M. Deiong J. M. Forney C. F. Prange R. (1999). Improving the thiobarbituric acid-recative–substances assay for estimating lipid peroxidation in plant tissues containing anthocyanin and other interfering compounds. Planta 207, 604–611. 10.1007/s004250050524 28456836

[B17] Hu G. Liu Y. Zhang X. Yao F. Huang Y. Ervin E. H.. (2015). Physiological evaluation of alkali-salt tolerance of thirty switchgrass (*Panicum virgatum*) lines. PLoS ONE 10:e0125305. 10.1371/journal.pone.0125305 26146987 PMC4492678

[B18] Hu L. Hu T. Zhang X. Pang H. Fu J. (2012). Exogenous glycine betaine ameliorates adverse effects of salt stress on perennial ryegrass. J. Amer. Soc. Hort. Sci. 137, 38–46.

[B19] Hu T. Yi H. Hu L. Fu J. (2013). Stomatal and metabolic limitations to photosynthesis resulting from NaCl stress in perennial ryegrass genotypes differing in salt tolerance. J. Amer. Soc. Hort. Sci. 138, 350–357.

[B20] Huang B. DaCosta M. Jiang Y. (2014). Research advances in mechanisms of grass tolerance to abiotic stressL from physiology to molecular biology. Crit. Rev. Plant Sci. 33, 141–189. 10.1080/07352689.2014.870411

[B21] Jiang Y. Tang J. Yu X. Camberato J. (2013). Growth and physiological responses of diverse perennial ryegrass accessions to increasing salinity, in 2012 Annual Reports in - Purdue University Turfgrass Science Program (West Lafayette, IN), 7–11.

[B22] Jones J. B. Wolf J. B. Mills H. A. (1991). Plant Analysis Handbook. Athens: Micro-Macro Publishing, Inc.

[B23] Khan M. I. R. Iqbal N. Masood A. Khan N. A. (2012). Variation in salt tolerance of wheat cultivars: role of glycinebetaine and ethylene. Pedosphere 22, 746–754. 10.1016/S1002-0160(12)60060-5

[B24] Khan M. I. R. Iqbal N. Masood A. Per T. S. Khan N. A. (2013). Salicylic acid alleviates adverse effects of heat stress on photosynthesis through changes in proline production and ethylene formation. Plant Signal. Behav. 8:e26374. 10.4161/psb.26374 24022274 PMC4091357

[B25] Kim J. Liu Y. Zhang X. Zhao B. Childs K. (2016). Analysis of salt-induced physiological and proline changes in 46 switchgrass (*Panicum virgatum*) lines indicates multiple responses modes. Plant Physiol. Biochem. 105, 203–212. 10.1016/j.plaphy.2016.04.020 27111258

[B26] Llanes A. Andrade A. Alenabo S. Luna V. (2016). Alterations of endogenous hormonal levels in plants under drought and salinity. Am. J. Plant Sci. 7, 1357–1371. 10.4236/ajps.2016.79129

[B27] Maggio A. Barbieri G. Raimondi G. Pascale S. (2010). Contrasting effects of GA3 treatments on tomato plants exposed to increasing salinity. J. Plant Growth Regul. 29, 63–72. 10.1007/s00344-009-9114-7

[B28] Man D. Bao Y. X. Han L. B. Zhang X. (2011). Drought tolerance associated with proline and hormone metabolism in two tall fescue cultivars. HortScience 46, 1027–1032.

[B29] Marcum K. B. Pessarakli M. (2010). Salinity tolerance of ryegrass turf cultivars. Hortscience 45, 1882–1884.

[B30] Ogweno J. O. Song X. S. Shi K. Hu W. H. Mao W. H. Zhou Y. H. (2008). Brassinosteroids alleviate heat-induced inhibition of photosynthesis by increasing carboxylation efficiency and enhancing antioxidant systems in Lycopersicon esculentum. J. Plant Growth Regul. 27, 49–57. 10.1007/s00344-007-9030-7

[B31] Ozdemir F. Bor M. Demiral T. Turkan I. (2004). Effects of 24-epibrassinolide on seed germination, seedling growth, lipid peroxidation, proline content and antioxidant system of rice (*Oryza sativa* L.) under salt stress. Plant Growth Regul. 42, 203–211. 10.1023/B:GROW.0000026509.25995.13

[B32] Ryu H. Cho Y. (2015). Plant hormones in salt stress tolerance. J. Plant Sci. 58, 147–155. 10.1007/s12374-015-0103-z

[B33] Shahbaz M. Ashraf M. (2007). Influence of exogenous application of brassinosteriods on growth and mineral nutrients of wheat (*Triticum aestivum* L.) under saline conditions Pak. J. Bot. 39, 513–522.

[B34] Shahbaz M. Ashraf M. Athar H. (2008). Does exogenous application of 24-epibrassinolide ameliorate salt induced growth inhibition in wheat (*Triticum aestivum* L.)? Plant Growth Regul. 55, 51–64. 10.1007/s10725-008-9262-y

[B35] Shahid M. A. Pervez M. A. Balal R. M. Mattson N. S. Rashid A. Ahmad R. (2011). Brassinosteriod (24-epibrassinolide) enhances growth and alleviates the deleterious effects induced by salt stress in pea (*Pisum sativum* L.). Aust. J. Crop Sci. 5, 500–510.

[B36] Shang Q. Song S. Zhang Z. Guo S. (2006). Exogenous brassinosteriod induced salt resistance of cucumber (*Cucumis sativus* L.) seedlings. Sci. Agric. Sinica 39, 1872–1877.

[B37] Sharma I. Ching E. Saini S. Bhardwaj R. Pati P. K. (2013). Exogenous application of brassinosteriod offers tolerance to salinity by altering stress responses in rice variety Pusa Basmati-1. Plant Physiol. Biochem. 69, 17–26. 10.1016/j.plaphy.2013.04.013 23707881

[B38] Shavrukov Y. Genc Y. Hayes J. (2012). The use of hydroponics in abiotic stress tolerance research, in Hydroponics- A Standard Methogology for Plant Biological Researches, ed Asao T. (Shanghai: InTech Press), 39–66.

[B39] Song W. Y. Yang H. C. Shao H. B. Zheng A. Z. Brestic M. (2014). The Alleviative effects of salicylic acid on the activities of catalase and superoxide dismutase in malting barley (*Hordeum uhulgare* L.) seedling leaves stressed by heavy metals. Clean Soil Air Water 42, 88–97. 10.1002/clen.201200310

[B40] Strivastava L. M. (2002). Plant Growth and Development: Hormones and Environment. San Diego, CA: Academic Press.

[B41] Sun S. An M. Han L. Yin S. (2015). Foliar application of 24-epibrassinolide improved salt stress tolerance of perennial ryegrass. HortScience 50, 1518–1523.

[B42] Tang X. Mu X. Shao H. Wang H. Brestic M. (2015). Global plant-responding mechanisms to salt stress: physiological and molecular levels and implications in biotechnology. Critic. Rev. Biotechnol. 35, 425–437. 10.3109/07388551.2014.889080 24738851

[B43] Vardhini B. Anjum N. A. (2015). Brassinosteriods make plant life easier under abiotic stresses mainly by modulating major components of antioxidant defense system. Front. Environ. Sci. 2:67 10.3389/fenvs.2014.00067

[B44] Vriet C. Russinova E. Reuzeau C. (2012). Boosting crop yields with plant steroids. Plant Cell Online 24, 842–857. 10.1105/tpc.111.094912 22438020 PMC3336137

[B45] Zdemir F. Bor M. Demiral T. Türkan S. (2004). Effects of 24-epibrassinolide on seed germination, seedling growth, lipid peroxidation, proline content and antioxidative system of rice (*Oryza sativa* L.) under salinity stress. Plant Growth Regul. 42, 203–211. 10.1023/B:GROW.0000026509.25995.13

[B46] Zhang X. Ervin E. H. Evanylo G. K. Haering K. C. (2009). Impact of biosolids on hormone metabolism in drought-stresses tall fescue. Crop Sci. 49, 364–370.

[B47] Zhang X. Ervin E. H. Liu Y. Hu G. Shang C. Fukao T. (2015). Differential responses of antioxidants, abscisic acid, and auxin to deficit irrigation in two perennial ryegrass cultivars contrasting in drought tolerance. J. Am. Soc. Hort. Sci. 140, 562–572.

[B48] Zhang X. Ervin E. H. Schmidt R. E. (2005). The role of leaf pigment and antioxidant levels in UV-B resistance of dark_ and light_green kentucky bluegrass. J. Am. Soc. Hort. Sci. 130, 836–841.

